# Crystal structure of (*R*,*S*)-2-hy­droxy-4-(methyl­sulfan­yl)butanoic acid

**DOI:** 10.1107/S2056989020003138

**Published:** 2020-03-17

**Authors:** Thomas P. Mawhinney, Yiyi Li, Deborah L. Chance, Steven P. Kelley, Valeri V. Mossine

**Affiliations:** a Department of Biochemistry, University of Missouri, Columbia, MO 65211, USA; b Experiment Station Chemical Laboratories, University of Missouri, Columbia, MO 65211, USA; c Department of Molecular Microbiology and Immunology, University of Missouri, Columbia, MO 65211, USA; d Department of Chemistry, University of Missouri, Columbia, MO 65211, USA

**Keywords:** crystal structure, me­thio­nine hy­droxy analog, 2-hy­droxy-4-(methyl­sulfan­yl)butanoic acid, HMTBA, CAS 583–91-5, hydrogen bonding

## Abstract

The title compound, a major animal feed supplement, abbreviated as HMTBA and alternatively called dl-me­thio­nine hy­droxy analogue, C_5_H_10_O_3_S, (**I**), was isolated in pure anhydrous monomeric form. The melting point is 302.5 K and the compound crystallizes in the monoclinic space group *P*2_1_/*c*, with two conformationally non-equivalent mol­ecules [(**I** ***A***) and (**I** ***B***)] in the asymmetric unit. The crystal structure is formed by alternating polar and non-polar layers running along the ***bc*** plane and features an extensive hydrogen-bonding network within the polar layers. The Hirshfeld surface analysis revealed a significant contribution of non-polar H⋯H and H⋯S inter­actions to the packing forces for both mol­ecules.

## Abstract 2

Me­thio­nine hy­droxy analogue, a common poultry feed supplement, has been obtained in crystalline form for the first time. The asymmetric unit contains two conformationally unequal mol­ecules that are involved in a two-dimensional inter­molecular hydrogen-bonding network.

## Chemical context   

**Figure 1:**
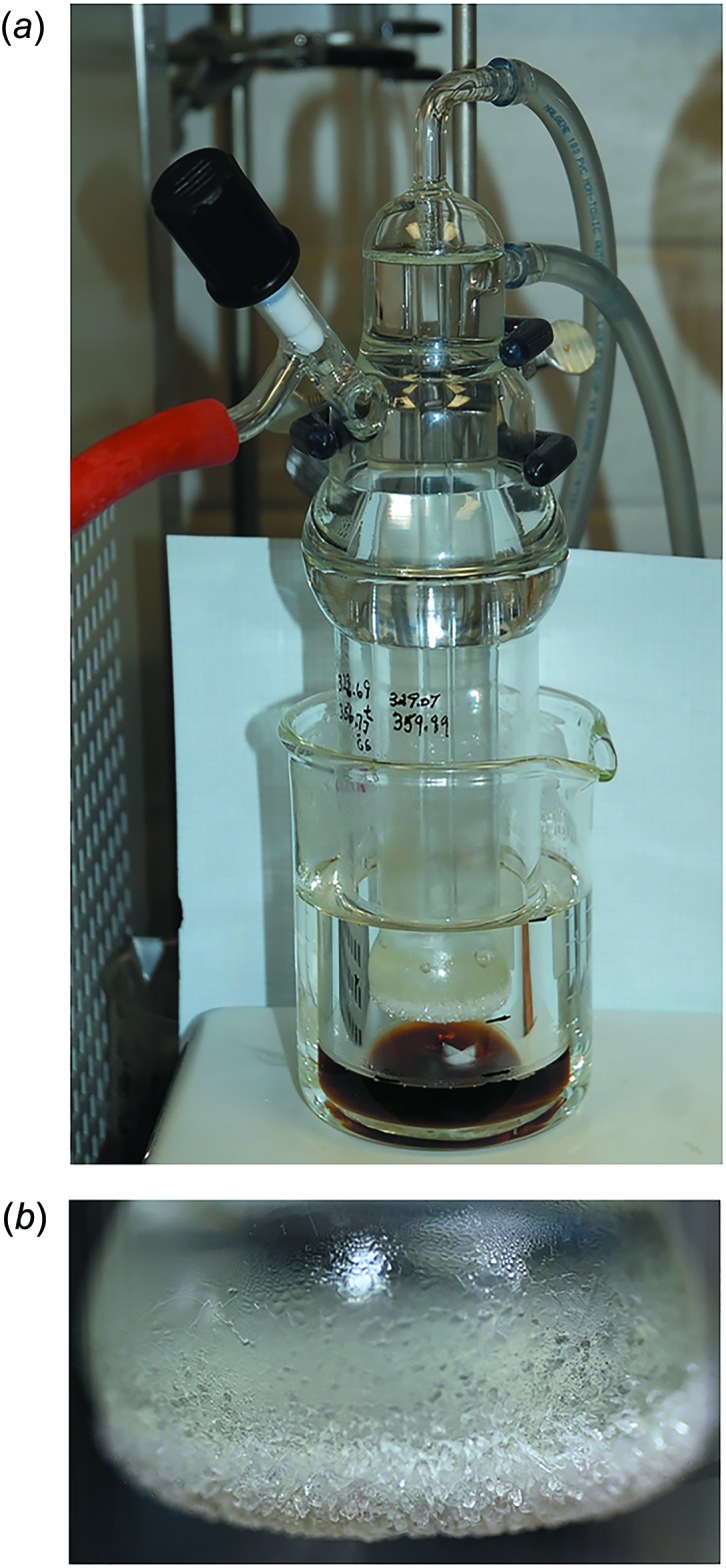
Preparation of crystals of (**I**). (*a*) Sublimation apparatus used for short-path distillation, (*b*) Crystals of (*R*,*S*)-HMTBA monomer formed on sublimator’s cold finger.

α-Hy­droxy carb­oxy­lic acids are indispensable players in plant and animal metabolism, and many of these substances are commercially important chemicals, because of their wide use in chemical industries and as pharmaceuticals, skin-care agents, or nutritional supplements (Bhalla *et al.*, 2013 ▸). 2-Hy­droxy-4-(methyl­sulfan­yl)butanoic acid (**I**) is a natural precursor in me­thio­nine biosynthesis, and, for decades, synthetic HMTBA has been used on an industrial scale as a supplement to animal feeds in order to boost me­thio­nine production, particularly in farmed poultry (Zhang *et al.*, 2015 ▸). In spite of its large-scale manufacture and use, commercial HMTBA is supplied as a brown, syrupy, racemic mixture, and it has not been reported to crystallize, even when isolated in chromatographically and enanti­omerically pure preparations (Busto *et al.*, 2014 ▸). One possible reason is that HMTBA readily forms dimeric and trimeric condensation products (Koban & Koberstein, 1984 ▸) which, along with the deliquescent behavior, may impede its crystallization. Crystal structures of free aliphatic α-hy­droxy carb­oxy­lic acids are rare, as a result of their propensity to oligomerize. Metal salts provide a means for stabilization of the α-hy­droxy carboxyl­ate monomers, and structures of two HMTBA metal salts, Cu[(*R*,*S*)-HMTBA]_2_ (CCDC 1018852; Yang *et al.*, 2015 ▸) and Zn[(*R*,*S*)-HMTBA]_2_ (CCDC 671417; Predieri *et al.*, 2009 ▸), have been solved by X-ray diffraction. In our attempts to separate monomeric and oligomeric forms of HMTBA, we have successfully isolated a high-purity crystalline sample of (**I**), shown in Fig. 1 ▸, and report here its characterization by X-ray diffraction.
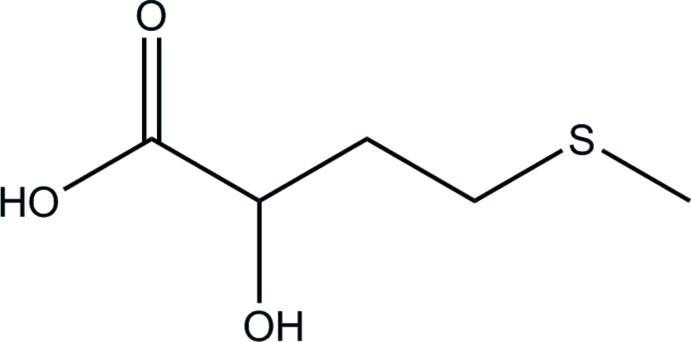


## Structural commentary   

**Figure 2:**
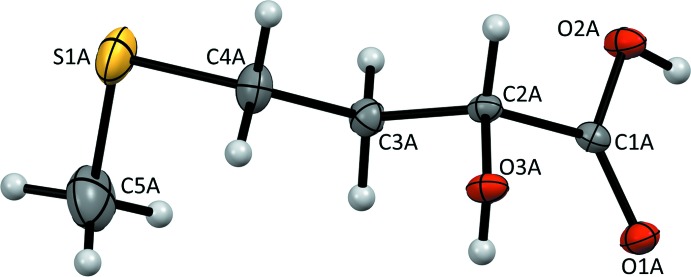
The atomic numbering and displacement ellipsoids at 50% probability level drawn for mol­ecule (**I** ***A***).

(*R*,*S*)-HMTBA crystallizes in the monoclinic space group *P*2_1_/*c*; the asymmetric unit consists of two mol­ecules in non-equivalent conformations, (**I** ***A***) and (**I** ***B***) (Table 1 ▸). The ORTEP views of the mol­ecules and numbering of the atoms are shown in Figs. 2 ▸ and 3 ▸. Bond lengths and valence angles in **(I)** are within ranges expected for the given structure. The C1–C5 backbone in (*R*)-(**I** ***A***) is in the *trans*, *trans*, *gauche+* (*t*, *t*, *g*+) conformation, with the chain of atoms C1*A* through S1*A* located in one plane [maximum deviation 0.144 (1) Å for C3*A*]. In the crystal of (*R*,*S*)-HMTBA copper salt (Yang *et al.*, 2015 ▸), a similar (*t*, *t*, *g*+) backbone rotamer exists in the (*S*)-HMTBA mol­ecule. Likewise, the respective (*t*, *t*, *g*+) conformation of the l-me­thio­nine side chain was found in the α-isoform of dl-Met crystal (CCDC 1028063; Görbitz *et al.*, 2014 ▸). The backbone conformation in (*R*)-(**I** ***B***) is the *gauche*+, *trans*, *gauche+* rotamer. An identical (*g*+, *t*, *g*+) conformation was adopted by (*R*)-HMTBA, mol­ecule *C*, which is coordinated to the zinc ion in the crystal of (*R*,*S*)-HMTBA zinc salt trihydrate (Predieri *et al.*, 2009 ▸). The conformation around the C1—C2 bond in (*R*)-(**I** ***B***) is close to eclipsed, in respect to the O1*B* and O3*B* atoms, with a O3*B*—C2*B*—C1*B*—O1*B* torsion angle of −10.81 (19)°. A similar spatial arrangement of the O1 and O3 atoms was reported in the aforementioned copper and zinc salts of HMTBA (Table 1 ▸), where simultaneous coordination of the carboxyl­ate and hydroxyl oxygen atoms to the metal ions provided for the formation of nearly flat five-membered chelate rings (Yang *et al.*, 2015 ▸; Predieri *et al.*, 2009 ▸). In crystal structures of the simplest α-hy­droxy carb­oxy­lic acids, glycolic acid (CCDC 1169248; Pijper, 1971 ▸) and l(+)-lactic acid (CCDC 1303177; Schouten *et al.*, 1994 ▸), the mol­ecular fragments including non-hydrogen atoms of the hydroxyl and carboxyl groups are also nearly flat (Table 1 ▸).

## Supra­molecular features   

**Figure 4:**
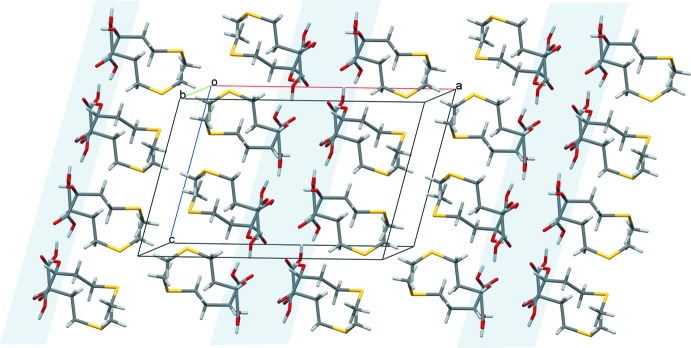
The mol­ecular packing in (**I**). Color code for crystallographic axes: red − *a*, green − *b*, blue − *c*. Highlighted are hydro­philic regions in the crystal.

**Figure 5:**
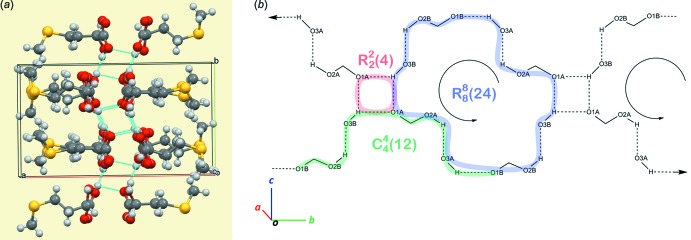
Hydrogen bonding in crystal structure of (**I**). (*a*) A view of the unit-cell contents shown in projection down the *a* axis. Hydrogen bonds are shown as cyan dotted lines. (*b*) Hydrogen-bonding patterns in the crystal structure of (**I**), as viewed down the *a* axis.

The crystal structure of (**I**) consists of alternating polar and non-polar sheets running along the *bc* plane (Fig. 4 ▸) and containing short O—H⋯O contacts within the polar layers (Fig. 4 ▸ and Table 2 ▸). Such a double-layered arrangement is typical for crystal structures of aliphatic l-α-amino acids and many other polar mol­ecules, and these are present in all reference structures of both HMTBA metal salts and me­thio­nine listed in Table 1 ▸. Within the polar sheets, the basic hydrogen-bonding pattern features infinite homodromic chains of hydrogen bonds spiraling along the *b-*axis direction (Fig. 5 ▸). The chains are linked through bifurcated hydrogen bonding that involves the hydroxyl O3*B*—H3*B* donor group and the carboxyl­ate O1*A* acceptor. One can recognize three basic motifs in the hydrogen-bonding pattern (in accordance with the topological notation system by Bernstein *et al.*, 1995 ▸): the 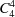
(12) motif forms homodromic infinite chains, which link similarly oriented mol­ecules; the small 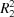
(4) ring and the large homodromic 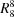
(24) ring, which are formed by the O3*B*—H3*B*⋯O1*A* links and the homodromic infinite chains that run along the *b* axis in opposite directions and are located on the opposite ‘half-sheets’ of the polar layer. The resulting pattern of conjugated rings is shown in Fig. 5 ▸ *b*: it represents one of two symmetrical, in respect to the twofold screw along the *b* axis, systems of hydrogen bonds that penetrate the polar layers.

**Figure 6:**
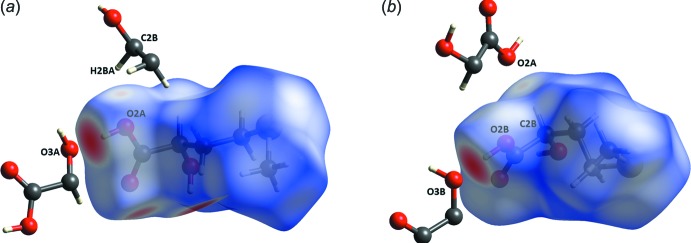
Views of the Hirshfeld surface for (*a*) mol­ecule (**I** ***A***) and (*b*) mol­ecule (**I** ***B***), mapped over the *d*_norm_ in the range 0.7691 to 1.1756 a.u. with the blue-to-red color palette reflecting distances from a point on the surface to the closest nuclei. The mol­ecular fragments involved in the shortest O—H⋯O and C—H⋯O inter­actions are shown.

**Figure 7:**
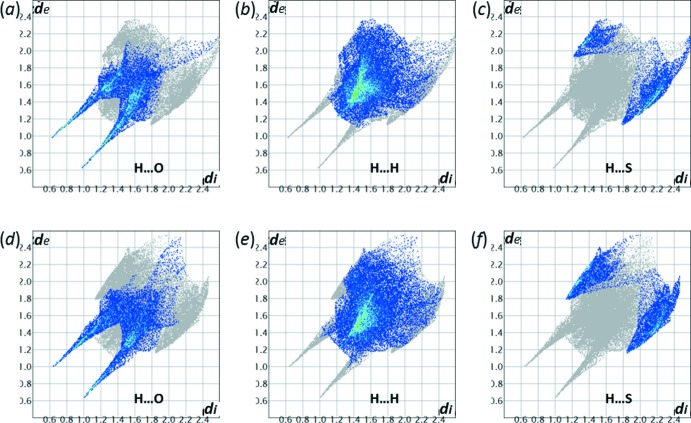
The two-dimensional fingerprint plots for (*a*)–(*c*) mol­ecule (**I** ***A***) and (*d*)–(*f*) mol­ecule (**I** ***B***), delineated into specific contacts: (*a*,*d*) O⋯H/H⋯O (32.3% and 28.5% contribution to the Hirshfeld surfaces of the respective mol­ecules); (*b*,*e*) H⋯H (48.9 and 50.4%); (*c*,*f*) H⋯S/S⋯H (14.3 and 18.2%).

In addition to the ‘classical’ O—H⋯O hydrogen bonds, there is one inter­molecular C2*B*—H*A*⋯O2*A* contact (Fig. 6 ▸ and Table 3 ▸) in the crystal structure of (**I**) that is shorter than the sum of the van der Waals radii. The Hirshfeld surface analysis (*CrystalExplorer17.5*; Spackman & Jayatilaka, 2009 ▸), however, reveals that the C—H⋯O contacts do not contribute significantly to the crystal packing forces, but that a major proportion, over 63% for (**I** ***A***) and over 68% for (**I** ***B***), of the inter­molecular contacts in the crystal structure of (**I**) is provided by non- or low-polar H⋯H and H⋯S inter­actions (Fig. 7 ▸ and Table 4 ▸). Compared to other aforementioned structures (Table 4 ▸), the relative contributions of the polar and non-polar inter­actions in (**I**) are similar to those found in HMTBA metal salts. The relative contribution of the polar component in me­thio­nine structures is somewhat higher, possibly because of the higher number of heteroatom-bonded hydrogen atoms, three, as compared to only two such protons present in mol­ecules of (**I**).

## Database survey   

Search of SciFinder, Google Scholar, and the Cambridge Structural Database (version 5.40, 2019 data update 3; Groom *et al.*, 2016 ▸), by both structure and chemical names, revealed no previous structural description of 2-hy­droxy-4-(methyl­sulfan­yl)butanoic acid in the solid state. Only two HMTBA structures, both of which are metal salts, Cu[(*R*,*S*)-HMTBA]_2_ (CCDC 1018852, Yang *et al.*, 2015 ▸) and Zn[(*R*,*S*)-HMTBA]_2_ (CCDC 671417, Predieri *et al.*, 2009 ▸), have been reported. The most closely related structure to (**I**) is me­thio­nine, for which a number of crystallographic studies have been published and these are referenced in Table 1 ▸. In addition to the structural features outlined in Tables 1 ▸ and 4 ▸, other similarities to (**I**) include l-me­thio­nine crystallizing in the monoclinic space group *P*2_1_ (CCDC 1207980, LMETON02; CCDC 1207981, LMETON10; Torii & Iitaka, 1973 ▸; Dalhus & Görbitz, 1996 ▸). The asymmetric unit in the crystal structure of l-Met also contains two conformationally unequal mol­ecules.

## Synthesis and crystallization   

Purely monomeric HMTBA in its free acid form is not commercially available because of the known propensity of α-hy­droxy carb­oxy­lic acids to oligomerize when concentrated (Koban & Koberstein, 1984 ▸); thus, we have evaluated the composition of a commercially available (*R*,*S*)-2-hy­droxy-4-(methyl­sulfan­yl)­butanoic acid (TCI America) as having 65-72% HMTBA monomer, 2.7–4.5% of its linear dimer, 0.14–0.35% of the linear trimer, and 28–35% water. A pure, anhydrous sample of racemic HMTBA monomer was prepared by employing a mild, short-path distillation technique that utilizes a sublimation apparatus (Fig. 1 ▸ *a*), half submerged in an ethyl­ene glycol bath that was maintained at 383 K. After 72 h, while under vacuum (10 torr) and the cold finger kept at 277 K, large colorless prisms of neat (**I**) were formed on the sublimator’s condenser (Fig. 1 ▸ *b*), which melted at 302.5 K.

## Refinement   

Crystal data, data collection and structure refinement details are summarized in Table 5 ▸. O-bound H atoms were located from the difference map and those bonded to C were placed in calculated positions. The coordinates of all H atoms were refined freely while the thermal parameters were constrained to ride on the carrier atoms, *U*_iso_(H) = 1.2–1.5*U*_eq_(C,O).

## Supplementary Material

Crystal structure: contains datablock(s) I. DOI: 10.1107/S2056989020003138/lh5949sup1.cif

Structure factors: contains datablock(s) I. DOI: 10.1107/S2056989020003138/lh5949Isup2.hkl

Supporting information file. DOI: 10.1107/S2056989020003138/lh5949Isup3.cml

CCDC reference: 1979735

Additional supporting information:  crystallographic information; 3D view; checkCIF report

## Figures and Tables

**Figure 3:**
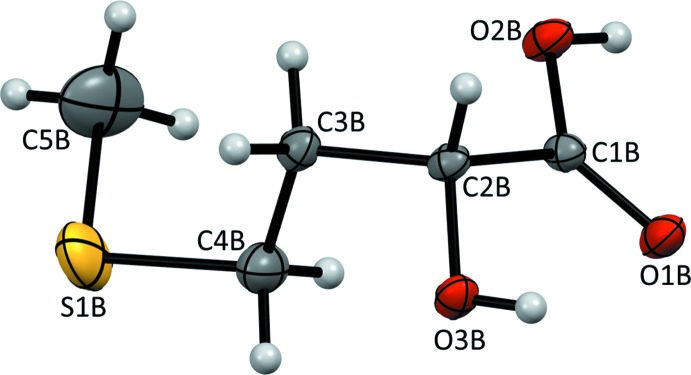
The atomic numbering and displacement ellipsoids at 50% probability level drawn for mol­ecule (**I** ***B***).

**Table 1:** Selected torsion angles (°) in (**I**) and related structures

	C1—C2—C3—C4	C2—C3—C4—S1	C3—C4—S1—C5	O1—C1—C2—O3/N1^*b*^	Ref.
(**I** ***A***)^*a*^	179.4 (1)	−164.2 (1)	−62.2 (2)	−27.8 (2)	This work
(**I** ***B***)^*a*^	−62.6 (2)	−178.2 (1)	−69.8 (2)	10.8 (2)	This work
Cu(HMTBA)_2_^*a*^	175.0 (4)	164.9 (3)	63.2 (5)	−14.2 (5)	(Yang *et al.*, 2015 ▸)
Zn(HMTBA)_2_: mol­ecule *A* (*S*)	−60.6 (7)	−157.5 (4)	−58.8 (6)	3.9 (6)	(Predieri *et al.*, 2009 ▸)
mol­ecule *B* (*R*)	64.6 (7)	−76.7 (7)	−68.2 (7)	9.5 (6)	
mol­ecule *C* (*R*)	60.0 (7)	173.4 (5)	66.2 (7)	9.7 (6)	
mol­ecule *D* (*S*)	−57.7 (9)	−174.7 (6)	−122.9 (8)	−1.3 (7)	
L-Met: mol­ecule *A*	71.8 (3)	171.6 (3)	−178.5 (3)	−16.3 (2)	(Dalhus & Görbitz, 1996 ▸)
mol­ecule B	74.1 (3)	71.5 (3)	72.4 (3)	−32.4 (2)	
α-DL-Met ^*a*^	−178.0 (2)	176.7 (2)	69.4 (3)	−29.4 (3)	(Görbitz *et al.*, 2014 ▸)
β-DL-Met ^*a*^	−173.6 (2)	−179.2 (1)	−175.0 (2)	−32.6 (2)	(Alagar *et al.*, 2005 ▸)
L-(+)-lactic acid				7.6 (1)	(Schouten *et al.*, 1994 ▸)
glycolic acid: mol­ecule *A*				−6.16 (2)	(Pijper, 1971 ▸)
mol­ecule *B*				−2.93 (2)	

Notes: (*a*) Signs of the angle values are given for the (*S*)-enanti­omer; (*b*) N1 in me­thio­nine.

**Table 2:** Hydrogen-bond geometry (Å, °)

*D*—H⋯*A*	*D*—H	H⋯*A*	*D*⋯*A*	*D*—H⋯*A*
O3*A*—H3*A*⋯O1*B*	0.736 (19)	2.013 (19)	2.7044 (14)	156.4 (19)
O3*B*—H3*B*⋯O1*A*	0.77 (2)	2.246 (19)	2.8480 (14)	135.6 (18)
O3*B*—H3*B*⋯O1*A* ^i^	0.77 (2)	2.325 (19)	2.9048 (14)	132.9 (18)
O2*A*—H2*A*⋯O3*A* ^ii^	0.89 (2)	1.71 (2)	2.5995 (14)	172.7 (18)
O2*B*—H2*B*⋯O3*B* ^iii^	0.86 (2)	1.79 (2)	2.6493 (14)	172.6 (19)

Symmetry codes: (i) 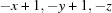; (ii) 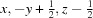; (iii) 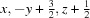.

**Table 3:** Suspected hydrogen bonds (Å, °)

*D*—H⋯*A*	*D*—H	H⋯*A*	*D*⋯*A*	*D*—H⋯*A*
C2*B*—H2*BA*⋯O2*A* ^i^	0.934 (17)	2.630 (17)	3.4068 (16)	141.1 (13)

Symmetry code: (i) *x*, 1 + *y*, *z*.

**Table 4:** Contributions (%) of inter­molecular atom–atom contacts to the Hirshfeld surface in (**I**) and related structures

	Backbone rotamer ^*a*^	H⋯H	S⋯H	O⋯H	Other			
HMTBA					O⋯O;	C⋯O;	C⋯H;	S⋯S
(**I** ***A***)	*ttg*−	48.9	14.3	32.3	1.7;	1.7;	0.9;	0.1
(**I** ***B***)	*g*−*tg*−	50.4	18.2	28.5	1.0;	1.7;	0.2;	0.1
								
					O⋯O;	C⋯O;	C⋯H;	Cu⋯O
Cu(HMTBA)_2_	*ttg*+	44.0	18.0	25.2	2.9;	1.7;	1.3;	6.9
								
Zn(HMTBA)_2_					O⋯O;	C⋯H;	S⋯S;	Zn⋯O
mol­ecule *A* (*S*)	*g*−*tg*−	48.4	18.4	22.0	2.4;	1.1;	0.3;	7.5
mol­ecule *B* (*R*)	*g*+*g*−*g*−	49.2	13.9	28.0	0.9;	1.0;	1.0;	4.9
mol­ecule *C* (*R*)	*g*+*tg*+	48.2	15.7	28.7	0.8;	0.8;	0.3;	5.0
								
L-Met					O⋯O;	C⋯O;	C⋯H;	C⋯C
mol­ecule *A*	*g*+*tt*	48.3	14.9	34.7	0.1;	0.6;	0.6;	0.5
mol­ecule *B*	*g* + *g*+*g+*	46.7	15.1	35.6	0.5;	0.6;	0.6;	0.5
								
β-DL-Met	*ttt*	48.7	14.6	35.6	0.3;	0.4;	1.3	

Note: (*a*) Refer to Table 1 ▸ for chirality of the mol­ecules and the actual torsion-angle values.

**Table 5:** Experimental details

Crystal data	
Chemical formula	C_5_H_10_O_3_S
*M*_r_	150.19
Crystal system, space group	Monoclinic, *P*2_1_/*c*
Temperature (K)	100
*a*, *b*, *c* (Å)	16.0940 (14), 8.8747 (8), 10.558 (1)
β (°)	105.654 (3)
*V* (Å^3^)	1452.1 (2)
*Z*	8
Radiation type	Mo *K*α
μ (mm^−1^)	0.38
Crystal size (mm)	0.34 × 0.25 × 0.07

Data collection	
Diffractometer	Bruker VENTURE CMOS area detector
Absorption correction	Multi-scan (*AXScale*; Bruker, 2017 ▸)
*T*_min_, *T*_max_	0.653, 0.746
No. of measured, independent and observed [*I* > 2σ(*I*)] reflections	37191, 4437, 3474
*R*_int_	0.071
(sin θ/λ)_max_ (Å^−1^)	0.715

Refinement	
*R*[*F* ^2^ > 2σ(*F* ^2^)], *wR*(*F* ^2^), *S*	0.044, 0.106, 1.05
No. of reflections	4437
No. of parameters	223
H-atom treatment	Only H-atom coordinates refined
Δρ_max_, Δρ_min_ (e Å^−3^)	0.53, −0.47

Computer programs: *APEX3* and *SAINT* (Bruker, 2017 ▸), *SHELXT2014* (Sheldrick, 2015*a* ▸), *SHELXL2017* (Sheldrick, 2015*b* ▸), *Mercury* (Macrae *et al.*, 2020 ▸), *OLEX2* (Dolomanov *et al.*, 2009 ▸) and *publCIF* (Westrip, 2010 ▸).

## supplementary crystallographic information

### Crystal data

**Table:**

C_5_H_10_O_3_S	*F*(000) = 640
*M**_r_* = 150.19	*D*_x_ = 1.374 Mg m^−^^3^
Monoclinic, *P*2_1_/*c*	Mo *K*α radiation, λ = 0.71073 Å
*a* = 16.0940 (14) Å	Cell parameters from 9911 reflections
*b* = 8.8747 (8) Å	θ = 2.6–30.5°
*c* = 10.558 (1) Å	µ = 0.38 mm^−^^1^
β = 105.654 (3)°	*T* = 100 K
*V* = 1452.1 (2) Å^3^	Plate, colourless
*Z* = 8	0.34 × 0.25 × 0.07 mm

### Data collection

**Table:**

Bruker VENTURE CMOS area detector diffractometer	3474 reflections with *I* > 2σ(*I*)
Radiation source: Incoatec IMuS microfocus Mo tube	*R*_int_ = 0.071
shutterless ω and phi scans	θ_max_ = 30.6°, θ_min_ = 2.6°
Absorption correction: multi-scan (*AXScale*; Bruker, 2017)	*h* = −22→23
*T*_min_ = 0.653, *T*_max_ = 0.746	*k* = −12→12
37191 measured reflections	*l* = −15→13
4437 independent reflections	

### Refinement

**Table:**

Refinement on *F*^2^	Primary atom site location: dual
Least-squares matrix: full	Secondary atom site location: difference Fourier map
*R*[*F*^2^ > 2σ(*F*^2^)] = 0.044	Hydrogen site location: mixed
*wR*(*F*^2^) = 0.106	Only H-atom coordinates refined
*S* = 1.05	*w* = 1/[σ^2^(*F*_o_^2^) + (0.0433*P*)^2^ + 0.6797*P*] where *P* = (*F*_o_^2^ + 2*F*_c_^2^)/3
4437 reflections	(Δ/σ)_max_ = 0.001
223 parameters	Δρ_max_ = 0.53 e Å^−^^3^
0 restraints	Δρ_min_ = −0.47 e Å^−^^3^

### Special details

**Table:**

Geometry. All esds (except the esd in the dihedral angle between two l.s. planes) are estimated using the full covariance matrix. The cell esds are taken into account individually in the estimation of esds in distances, angles and torsion angles; correlations between esds in cell parameters are only used when they are defined by crystal symmetry. An approximate (isotropic) treatment of cell esds is used for estimating esds involving l.s. planes.

### Fractional atomic coordinates and isotropic or equivalent isotropic displacement parameters (Å^2^)

**Table:**

	*x*	*y*	*z*	*U*_iso_*/*U*_eq_	
S1A	0.84095 (3)	0.20993 (6)	0.52925 (4)	0.03405 (12)	
S1B	0.92449 (3)	0.76333 (6)	0.21950 (6)	0.04228 (14)	
O3A	0.55286 (6)	0.36868 (11)	0.33141 (9)	0.0159 (2)	
H3A	0.5659 (12)	0.439 (2)	0.3057 (18)	0.024*	
O1A	0.53671 (6)	0.35894 (11)	0.06723 (9)	0.0184 (2)	
O3B	0.64397 (7)	0.61637 (11)	0.07733 (10)	0.0179 (2)	
H3B	0.5982 (13)	0.587 (2)	0.0729 (19)	0.027*	
O2A	0.56541 (7)	0.11169 (10)	0.08154 (10)	0.0175 (2)	
H2A	0.5570 (11)	0.123 (2)	−0.005 (2)	0.026*	
O2B	0.66367 (7)	0.88568 (11)	0.33618 (10)	0.0205 (2)	
H2B	0.6535 (12)	0.880 (2)	0.412 (2)	0.031*	
O1B	0.60884 (7)	0.65325 (11)	0.31386 (10)	0.0217 (2)	
C2A	0.59189 (8)	0.24683 (14)	0.28125 (13)	0.0131 (2)	
H2AA	0.5714 (10)	0.1576 (19)	0.3142 (16)	0.016*	
C1A	0.56017 (8)	0.24609 (14)	0.13230 (13)	0.0134 (2)	
C3B	0.74743 (9)	0.81369 (16)	0.13945 (14)	0.0183 (3)	
H3BA	0.7508 (11)	0.820 (2)	0.0534 (18)	0.022*	
H3BB	0.7552 (11)	0.914 (2)	0.1762 (17)	0.022*	
C2B	0.65613 (9)	0.76258 (14)	0.13541 (13)	0.0150 (2)	
H2BA	0.6158 (11)	0.8295 (19)	0.0850 (16)	0.018*	
C3A	0.69066 (9)	0.25339 (16)	0.32158 (13)	0.0173 (3)	
H3AA	0.7133 (11)	0.167 (2)	0.2847 (17)	0.021*	
H3AB	0.7095 (11)	0.340 (2)	0.2816 (17)	0.021*	
C4A	0.72730 (10)	0.25292 (19)	0.47077 (15)	0.0231 (3)	
H4AA	0.7001 (12)	0.174 (2)	0.5080 (18)	0.028*	
H4AB	0.7168 (12)	0.347 (2)	0.5062 (18)	0.028*	
C4B	0.81668 (10)	0.70791 (19)	0.21803 (19)	0.0280 (3)	
H4BA	0.8093 (12)	0.610 (2)	0.1778 (19)	0.034*	
H4BB	0.8140 (12)	0.696 (2)	0.307 (2)	0.034*	
C5A	0.88873 (13)	0.3629 (3)	0.4614 (2)	0.0441 (5)	
H5AA	0.8738 (17)	0.356 (3)	0.359 (3)	0.066*	
H5AB	0.9499 (17)	0.348 (3)	0.497 (3)	0.066*	
H5AC	0.8699 (16)	0.455 (3)	0.489 (3)	0.066*	
C5B	0.93633 (15)	0.9292 (3)	0.3193 (3)	0.0578 (7)	
H5BA	0.898 (2)	1.008 (4)	0.280 (3)	0.087*	
H5BB	0.994 (2)	0.959 (3)	0.336 (3)	0.087*	
H5BC	0.9255 (19)	0.895 (3)	0.407 (3)	0.087*	
C1B	0.64041 (8)	0.75921 (14)	0.27099 (13)	0.0151 (2)	

### Atomic displacement parameters (Å^2^)

**Table:**

	*U*^11^	*U*^22^	*U*^33^	*U*^12^	*U*^13^	*U*^23^
S1A	0.02048 (19)	0.0455 (3)	0.0314 (2)	0.00432 (17)	−0.00131 (16)	0.00794 (19)
S1B	0.0198 (2)	0.0453 (3)	0.0635 (3)	−0.00015 (17)	0.0142 (2)	−0.0085 (2)
O3A	0.0236 (5)	0.0107 (4)	0.0148 (5)	0.0014 (4)	0.0076 (4)	0.0001 (4)
O1A	0.0248 (5)	0.0136 (4)	0.0156 (5)	0.0012 (4)	0.0035 (4)	0.0024 (4)
O3B	0.0208 (5)	0.0159 (5)	0.0179 (5)	−0.0047 (4)	0.0067 (4)	−0.0036 (4)
O2A	0.0291 (5)	0.0118 (4)	0.0122 (5)	0.0001 (4)	0.0063 (4)	−0.0007 (4)
O2B	0.0331 (6)	0.0147 (5)	0.0154 (5)	−0.0045 (4)	0.0097 (4)	−0.0017 (4)
O1B	0.0305 (5)	0.0159 (5)	0.0219 (5)	−0.0040 (4)	0.0126 (4)	0.0002 (4)
C2A	0.0176 (6)	0.0100 (5)	0.0119 (6)	0.0006 (4)	0.0044 (5)	−0.0002 (4)
C1A	0.0142 (6)	0.0119 (6)	0.0148 (6)	−0.0017 (4)	0.0050 (5)	−0.0002 (5)
C3B	0.0209 (6)	0.0180 (6)	0.0170 (7)	−0.0025 (5)	0.0070 (5)	0.0005 (5)
C2B	0.0192 (6)	0.0130 (6)	0.0127 (6)	−0.0002 (5)	0.0045 (5)	0.0011 (5)
C3A	0.0184 (6)	0.0185 (6)	0.0148 (6)	−0.0003 (5)	0.0043 (5)	−0.0005 (5)
C4A	0.0193 (7)	0.0309 (8)	0.0175 (7)	0.0011 (6)	0.0021 (5)	−0.0003 (6)
C4B	0.0212 (7)	0.0227 (7)	0.0392 (10)	−0.0007 (6)	0.0067 (7)	0.0013 (7)
C5A	0.0252 (9)	0.0575 (13)	0.0474 (12)	−0.0085 (9)	0.0058 (8)	0.0033 (10)
C5B	0.0321 (10)	0.0452 (12)	0.0835 (19)	−0.0106 (9)	−0.0059 (11)	−0.0134 (12)
C1B	0.0170 (6)	0.0137 (6)	0.0143 (6)	0.0006 (5)	0.0040 (5)	0.0005 (5)

### Geometric parameters (Å, º)

**Table:**

S1A—C5A	1.801 (2)	C3B—C2B	1.5273 (19)
S1A—C4A	1.8063 (15)	C3B—H3BA	0.926 (18)
S1B—C5B	1.791 (3)	C3B—H3BB	0.967 (17)
S1B—C4B	1.7995 (16)	C2B—C1B	1.5197 (19)
O3A—C2A	1.4227 (15)	C2B—H2BA	0.934 (17)
O3A—H3A	0.736 (19)	C3A—C4A	1.526 (2)
O1A—C1A	1.2155 (16)	C3A—H3AA	0.974 (18)
O3B—C2B	1.4259 (16)	C3A—H3AB	0.968 (18)
O3B—H3B	0.77 (2)	C4A—H4AA	0.967 (19)
O2A—C1A	1.3197 (15)	C4A—H4AB	0.95 (2)
O2A—H2A	0.89 (2)	C4B—H4BA	0.96 (2)
O2B—C1B	1.3171 (16)	C4B—H4BB	0.95 (2)
O2B—H2B	0.86 (2)	C5A—H5AA	1.04 (3)
O1B—C1B	1.2131 (16)	C5A—H5AB	0.96 (3)
C2A—C1A	1.5167 (18)	C5A—H5AC	0.95 (3)
C2A—C3A	1.5318 (19)	C5B—H5BA	0.95 (3)
C2A—H2AA	0.959 (17)	C5B—H5BB	0.94 (3)
C3B—C4B	1.521 (2)	C5B—H5BC	1.03 (3)
			
C5A—S1A—C4A	101.91 (9)	C4A—C3A—H3AB	111.9 (10)
C5B—S1B—C4B	100.32 (10)	C2A—C3A—H3AB	109.5 (10)
C2A—O3A—H3A	108.2 (15)	H3AA—C3A—H3AB	105.0 (15)
C2B—O3B—H3B	110.7 (14)	C3A—C4A—S1A	115.28 (11)
C1A—O2A—H2A	108.0 (12)	C3A—C4A—H4AA	109.3 (11)
C1B—O2B—H2B	109.6 (13)	S1A—C4A—H4AA	103.5 (11)
O3A—C2A—C1A	109.29 (10)	C3A—C4A—H4AB	110.1 (11)
O3A—C2A—C3A	113.39 (11)	S1A—C4A—H4AB	108.8 (11)
C1A—C2A—C3A	108.79 (10)	H4AA—C4A—H4AB	109.5 (16)
O3A—C2A—H2AA	105.3 (10)	C3B—C4B—S1B	113.51 (11)
C1A—C2A—H2AA	108.7 (10)	C3B—C4B—H4BA	109.5 (12)
C3A—C2A—H2AA	111.3 (10)	S1B—C4B—H4BA	104.8 (11)
O1A—C1A—O2A	124.01 (12)	C3B—C4B—H4BB	112.4 (12)
O1A—C1A—C2A	123.28 (11)	S1B—C4B—H4BB	108.6 (12)
O2A—C1A—C2A	112.61 (11)	H4BA—C4B—H4BB	107.6 (17)
C4B—C3B—C2B	112.94 (12)	S1A—C5A—H5AA	111.3 (15)
C4B—C3B—H3BA	110.1 (11)	S1A—C5A—H5AB	104.0 (16)
C2B—C3B—H3BA	107.5 (11)	H5AA—C5A—H5AB	109 (2)
C4B—C3B—H3BB	110.7 (10)	S1A—C5A—H5AC	108.7 (16)
C2B—C3B—H3BB	107.7 (10)	H5AA—C5A—H5AC	112 (2)
H3BA—C3B—H3BB	107.7 (15)	H5AB—C5A—H5AC	112 (2)
O3B—C2B—C1B	110.41 (10)	S1B—C5B—H5BA	113.0 (18)
O3B—C2B—C3B	107.56 (11)	S1B—C5B—H5BB	106.3 (19)
C1B—C2B—C3B	112.52 (11)	H5BA—C5B—H5BB	112 (3)
O3B—C2B—H2BA	109.9 (10)	S1B—C5B—H5BC	105.4 (17)
C1B—C2B—H2BA	106.4 (10)	H5BA—C5B—H5BC	111 (3)
C3B—C2B—H2BA	110.0 (10)	H5BB—C5B—H5BC	108 (2)
C4A—C3A—C2A	111.71 (11)	O1B—C1B—O2B	123.66 (12)
C4A—C3A—H3AA	109.2 (10)	O1B—C1B—C2B	124.04 (12)
C2A—C3A—H3AA	109.3 (10)	O2B—C1B—C2B	112.30 (11)
			
O3A—C2A—C1A—O1A	27.77 (17)	C2A—C3A—C4A—S1A	164.16 (10)
C3A—C2A—C1A—O1A	−96.52 (15)	C5A—S1A—C4A—C3A	62.16 (15)
O3A—C2A—C1A—O2A	−155.72 (10)	C2B—C3B—C4B—S1B	178.17 (10)
C3A—C2A—C1A—O2A	79.99 (13)	C5B—S1B—C4B—C3B	69.76 (16)
C4B—C3B—C2B—O3B	−59.22 (16)	O3B—C2B—C1B—O1B	−10.81 (19)
C4B—C3B—C2B—C1B	62.59 (16)	C3B—C2B—C1B—O1B	−130.99 (14)
O3A—C2A—C3A—C4A	58.73 (15)	O3B—C2B—C1B—O2B	170.24 (11)
C1A—C2A—C3A—C4A	−179.44 (11)	C3B—C2B—C1B—O2B	50.06 (15)

### Hydrogen-bond geometry (Å, º)

**Table:**

*D*—H···*A*	*D*—H	H···*A*	*D*···*A*	*D*—H···*A*
O3*A*—H3*A*···O1*B*	0.736 (19)	2.013 (19)	2.7044 (14)	156.4 (19)
O3*B*—H3*B*···O1*A*	0.77 (2)	2.246 (19)	2.8480 (14)	135.6 (18)
O3*B*—H3*B*···O1*A*^i^	0.77 (2)	2.325 (19)	2.9048 (14)	132.9 (18)
O2*A*—H2*A*···O3*A*^ii^	0.89 (2)	1.71 (2)	2.5995 (14)	172.7 (18)
O2*B*—H2*B*···O3*B*^iii^	0.86 (2)	1.79 (2)	2.6493 (14)	172.6 (19)

Symmetry codes: (i) −*x*+1, −*y*+1, −*z*; (ii) *x*, −*y*+1/2, *z*−1/2; (iii) *x*, −*y*+3/2, *z*+1/2.
